# Risk communication and community engagement in the context of COVID-19 response in Bangladesh: a qualitative study

**DOI:** 10.3389/fpubh.2023.1267446

**Published:** 2024-01-05

**Authors:** Mohammed Kamruzzaman, Aminur Rahman, Daniel D. Reidpath, Sadika Akhter

**Affiliations:** ^1^Health Systems and Population Studies Division, ICDDR,B, Dhaka, Bangladesh; ^2^Institute for Global Health and Development, Queen Margaret University, Edinburgh, Scotland; ^3^School of Health and Social Development, Deakin University, Melbourne, VIC, Australia

**Keywords:** COVID-19, risk communication, community engagement, framing theory, qualitative research, Bangladesh

## Abstract

**Background:**

The global COVID-19 pandemic profoundly impacted nations worldwide, and Bangladesh was no exception. In response, the government of Bangladesh implemented community awareness initiatives aimed at containing the spread of the virus, aligned with international guidelines and recommendations. Despite these efforts, a lack of comprehensive community awareness programs played an essential role during the pandemic, not the preventive measures. A qualitative study employing framing theory was conducted to gain a deeper insight into how the social context influenced risk communication and community response throughout the COVID-19 pandemic in Bangladesh.

**Methods:**

The study was conducted in four selected districts of Bangladesh from February to May 2022 using complementary data collection methods, including key informant interviews, in-depth interviews, and focus group discussions with purposely selected participants. Data were analyzed thematically by following six steps of the thematic analysis process. Codes were developed based on the data and summarized into themes and sub-themes grounded on the codes.

**Results:**

The findings indicate that the government of Bangladesh, along with development partners and non-government organizations, made a significant effort to raise awareness about COVID-19 in the community. However, there were certain limitations to this effort. These include a lack of social science and public health approaches to understanding the pandemic; inadequate coordination among the authorities for COVID-19 prevention and control; technological and geographical barriers for disseminating messages; the living conditions and lack of facilities; socio-cultural norms in understanding the COVID-19 health messages, and the gendered understanding of the messages. The findings also revealed that the awareness activities remained a one-way approach to inform the people and faced challenges to actively engage and create ownership of the community in the pandemic response.

**Conclusion:**

The study identified gaps in implementing risk communication and community engagement strategies in Bangladesh during the COVID-19 pandemic. Increasing focus on public health and prioritizing community ownership is essential to designing a more effective community awareness campaign. This approach will help ensure that health messages are communicated effectively and tailored to different communities’ needs.

## Background

The novel coronavirus disease (COVID-19) has been a significant incident, with outcomes ranging from actual deaths to the collapse of the world economy (1, 2). Due to increased infections and fatalities, the World Health Organization (WHO), on March 11, 2020, declared it a global pandemic. In order to stop the pandemic, the WHO pleaded with governments to follow its recommendations, which include ordering people to “stay at home” and testing as many people as possible who had symptoms of COVID-19 (3).

Bangladesh was one of the countries affected by COVID-19. On March 8, 2020, the government of Bangladesh officially recognized the first COVID-19 case. From that point, the number of infections and fatalities steadily climbed, following the global trend (4). The government launched several programs to address the issue and to contain the pandemic. The initiatives included, among many others things, providing personal protective equipment (PPE) and other logistics for healthcare professionals and identifying and treating COVID-19 patients. The government also organized regular press briefings to inform people about the response to the pandemic and how to prevent and control infections and deaths (5, 6).

In order to contain the pandemic, the World Health Organization (WHO) strongly emphasized the importance of community engagement in its public health-related initiatives and recommendations. For instance, the COVID-19 strategy emphasizes community involvement in the risk communication (7). The strategy focuses on establishing preventive behaviors like hand washing, refraining from touching one’s face, practicing good respiratory hygiene, maintaining distance, isolating confirmed cases in a community facility or home, and cooperating with physical distancing measures and movement restrictions when necessary. Incorporating local input and circumstances into health care services, encouraging community-level education, and focusing on community empowerment and mobilization are other key components of the plan. Additionally, the WHO strategy strongly emphasized disseminating accurate information regarding hazards, the steps that should be taken by health authorities, and the precautions individuals could take^.^ (7) Considering the WHO priorities, The Government of Bangladesh also emphasized community engagement in its COVID-19 prevention and control activities.

The principle of community engagement or participation in Bangladesh’s health sector was a pre-COVID issue (e.g., Community Support Committee-CSC for hospital management) (8). It was reinforced during the pandemic because of the enormous public health concern worldwide (9).

The Government of Bangladesh prioritized community engagement in its national COVID-19-related initiatives and guidelines, in keeping with international recommendations and advice (10–13). The “Risk Communication and Community Engagement (RCCE)” portion of the “Bangladesh Preparedness and Response Plan for COVID-19” placed a strong emphasis on enabling people and communities to control the spread of COVID-19 by changing behavior and taking coordinated community action (13). The government, several development partners, and NGOs also raised public awareness by involving communities in preventing and controlling infections. They did this, for instance, by mandating mask use, hand hygiene, and social distancing. An NGO-led effort named “Community Resilience to Prevent Coronavirus” focused on educating local leaders, volunteers, and community groups while including them in awareness campaigns with the help of business, public, and non-governmental organization (NGO) partners (14). The effort produced communications material and messages of a wide variety and disseminated them through various media outlets, including radio and television, billboards, social media, local government officials, and religious leaders (15).

Despite these engagement efforts, the country’s overall COVID-19 status indicated low community awareness of the virus, contributing to higher infection rates in many locations–urban and rural (16, 17). A further investigation found that the delta variant of the virus, initially detected in India, had made its way into Bangladesh via the land border. Subsequently, during the Eid ul Fitr festival in May 2021, it spread extensively to other districts, primarily due to a lack of awareness among the general public (18).

A number of studies conducted in Bangladesh explored social and community perceptions regarding the prevention and control of coronavirus. One such study in rural Bangladesh revealed a lack of comprehensive knowledge about the spread, symptoms, and prevention of the virus (19). Additionally, there existed a disparity between community beliefs concerning the pandemic and the science-driven interventions advocated by health professionals (20). In another study focusing on social and cultural beliefs related to COVID-19 in Bangladesh, the critical role of community engagement was highlighted in ensuring the effective implementation of COVID-19 prevention programs (21).

The COVID-19 virus transmission is best prevented and controlled through vaccinations. Although there is always a chance of illness despite vaccination, the government of Bangladesh has made good progress in immunizing the population (22). These facts have led many specialists to underline the need for improved and expanded community awareness campaigns to limit coronavirus transmission (17). As COVID-19 continues to be a global concern and there is concern over a potential future wave, thorough implementation of the health safety requirements is also crucial (23).

Within this context, the primary objective of this study was to present comprehensive findings on community perceptions, preparedness, and responses to COVID-19 health guidelines. Additionally, the study aimed to assess awareness activities within selected districts in Bangladesh, providing valuable insights into the effectiveness of these initiatives.

## Framing theory

Through the lens of framing theory, we conducted a qualitative study to understand how the social context influences risk communication and community response during the COVID-19 pandemic (24, 25). Framing theory is founded on the basic assumption that an issue can be perceived from various perspectives and that people form opinions based on their previously held beliefs. Further, it refers to how people construct a particular understanding of an issue and act based on their perspective of the problem (24). In summary, framing theory emphasizes the importance of how information is provided to the community and the context in which it is presented. Both of these can significantly influence the community’s understanding, perceptions, and actions, especially in the domain of public health. Recognizing and understanding the power of context or frame is essential for governments, media organizations, and public health officials in effectively communicating and managing health crises and promoting healthier behaviors (24). Framing theory have been applied to various research areas in the field of health, including community response to vaccination, health communication, the use of alcohol, and sexual and reproductive health (25, 26). However, a comprehensive analysis of risk communication in Bangladesh throughout the pandemic has not yet been conducted. The Bangladesh Health Sector Response to an Emergency Plan for Novel Coronavirus recommended communication as one of the effective strategies for limiting the spread of viruses, and the lack of an evaluation is an issue.

## Methods

### Study design and site

In-depth interviews (IDI) and focus group discussions (FGD) were complementary data-gathering methods employed in this qualitative study. Bangladesh, a densely populated country middle-income country with an estimated population of 165 million, faced a serious challenge dealing with the COVID-19 pandemic.

This study focused on the collection of primary data from four purposively selected districts between February and May 2022, with a particular emphasis on three divisions that reported the highest COVID-19 cases: Dhaka (70.5%), Chattogram (12.8%), and Rajshahi (4.2%) divisions. Dhaka, the capital of Bangladesh, is known as having one of the largest and most densely populated slum areas. In the Dhaka district, the infection rate was 60.6%, highlighting the urgency of examining the dynamics of the pandemic in urban settings and in vulnerable urban communities in particular. Kishoreganj, situated in the Dhaka division, is characterized by wetlands and a hard-to-reach area due to its geographical features. The district has areas of high poverty, and investigating Kishoreganj provides insights into the challenges other similar regions faced during the pandemic. In the Chattogram division, Cox’s Bazaar is recognized as a popular tourist destination, which made it susceptible to the spread of the virus. This district’s inclusion in the study allowed for an examination of the pandemic’s impact on tourism-dependent regions. Under the Rajshahi division, the Pabna district emerged as one of the areas severely affected by the pandemic.

### Study sampling and participants

**Figure 1:**
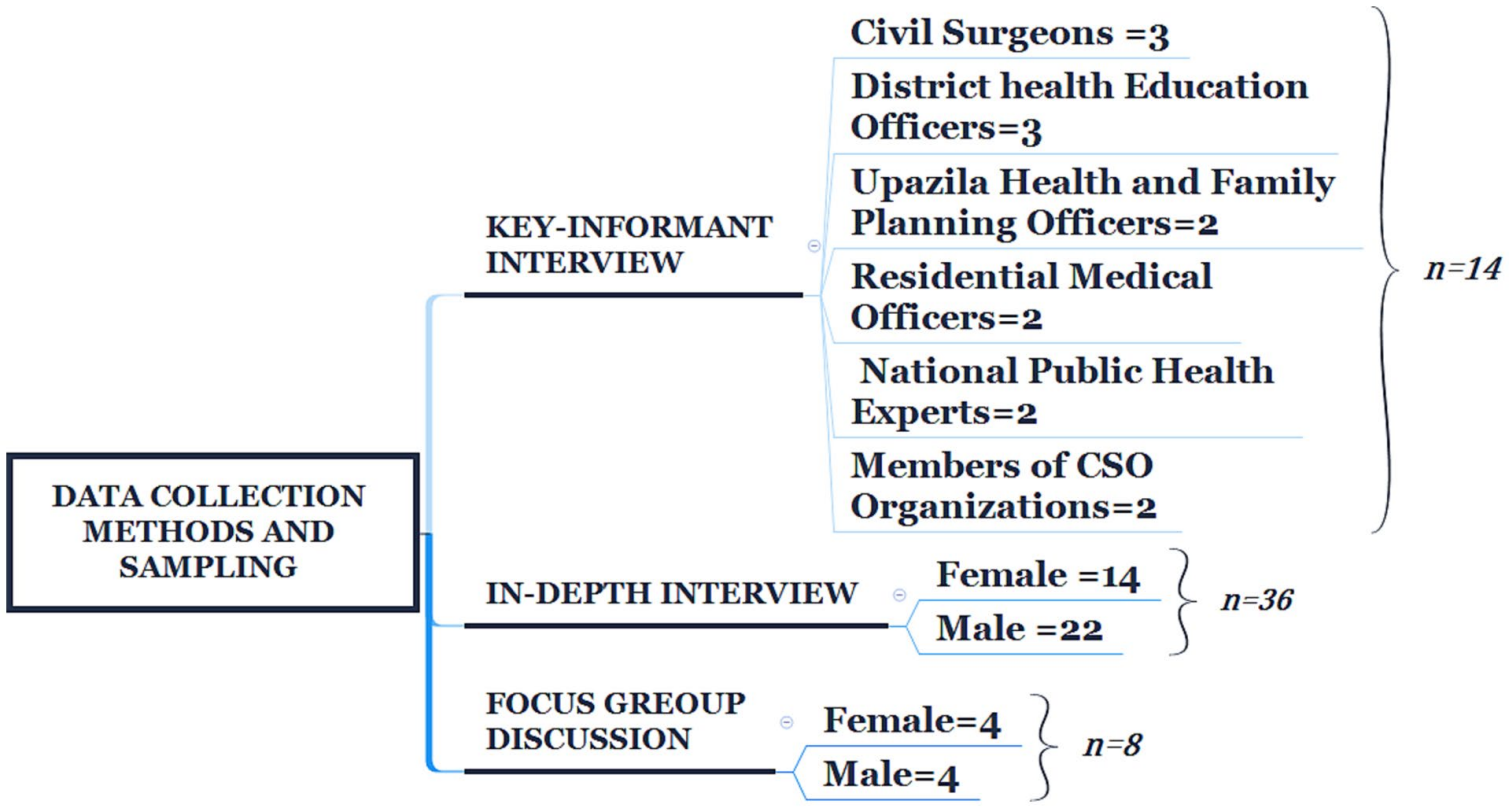
Data collection methods and sampling.

We conducted 36 IDIs, 14 KIIs, and 8 FGDs at various study sites (Figure 1). The KIIs included civil surgeons, district-level health education officers, sub-district-level health and family planning officers, residential medical officers, as well as local civil society organization (CSO) members and pertinent national specialists. These KIIs were chosen purposively. They were all involved in the prevention and control of COVID-19. The KIIs were used to investigate the government effort in response to the community awareness program during the COVID-19 pandemic, including the strategies for promoting good hygiene at the community level and coordination of the activities with ministries working during the emergency period. Aspects of the community awareness program and factors that encouraged community members to adopt preventive measures and obstacles to carrying out the program’s operations were investigated.

In-depth interviews were conducted with male (n = 22) and female caregivers (n = 14) in communities to explore actual knowledge, practice, drivers, and barriers to handwashing and use of facemasks. We purposely targeted both females and males for their professional responsibilities as care providers. The IDIs included males and females with diverse characteristics (farmers, daily laborers, transport workers, garment workers, and businesspersons) all of whom were exposed to different awareness messages related to COVID-19. The FGDs were conducted separately with males (n = 4) and females (n = 4) to capture the perception, preparedness, and response to COVID-19. Each FGD session consisted of eight participants, and there were a total of 64 participants in the eight FGDs. We purposely selected communicative residents who were available during the day to participate.

### Data collection procedure

For KIIs, IDIs, and FGDs, we developed separate open-ended interview guidelines, which were used to collect the data. The interviews focused on four major themes: (a) knowledge and practice regarding the COVID-19 awareness messages among the study participants; (b) facilities available within the public places and residences; (c) challenges to adherence to the preventive measures; and (d) suggestions and recommendations to meet their needs. The researchers used follow-up questions based on the interviewees’ responses to the initial open-ended questions to elicit more information about the respondents’ specific experiences. The combination of open-ended and probing questions facilitated more in-depth conversations between the researchers and the respondents.

Three qualified research officers with a background in Anthropology or Sociology were trained to conduct interviews in the local language—Bengali—under the supervision of the study’s principal investigator (SA). Before the beginning of data collection, a three-day “Research Methods Workshop” was organized. SA led the workshop for the investigation. It included (1) an overview of qualitative research methods, (2) practice undertaking interviews using open-ended guidelines, (3) writing transcripts, and (4) ethical procedures. The interviews (KIIs, IDIs, and FGDs) were conducted face-to-face at the respondents’ preferred times and locations. All interviews were recorded with a digital recorder. The fieldwork for data collection was conducted under the supervision of SA, who has academic training and experience conducting qualitative research. The researcher (MK) oversaw the data collection from the field, including conducting the KIIs at all field sites. One researcher (SA) moderated the FGDs, while another (MK) took comprehensive notes alongside the FGD recordings.

The steps in preparation for the field research included pre-testing and adaptation/revision of the study data collection instruments (27). During the pre-testing, the interviewers kept a record of their experiences in the field—what they observed during the interviews—and shared it in the debriefing session for the team’s learning. This procedure helped to assess and review the mode of conducting interviews and discussions with the respondents and to identify any biases during the interviews.

### Data analysis

**Figure 2:**
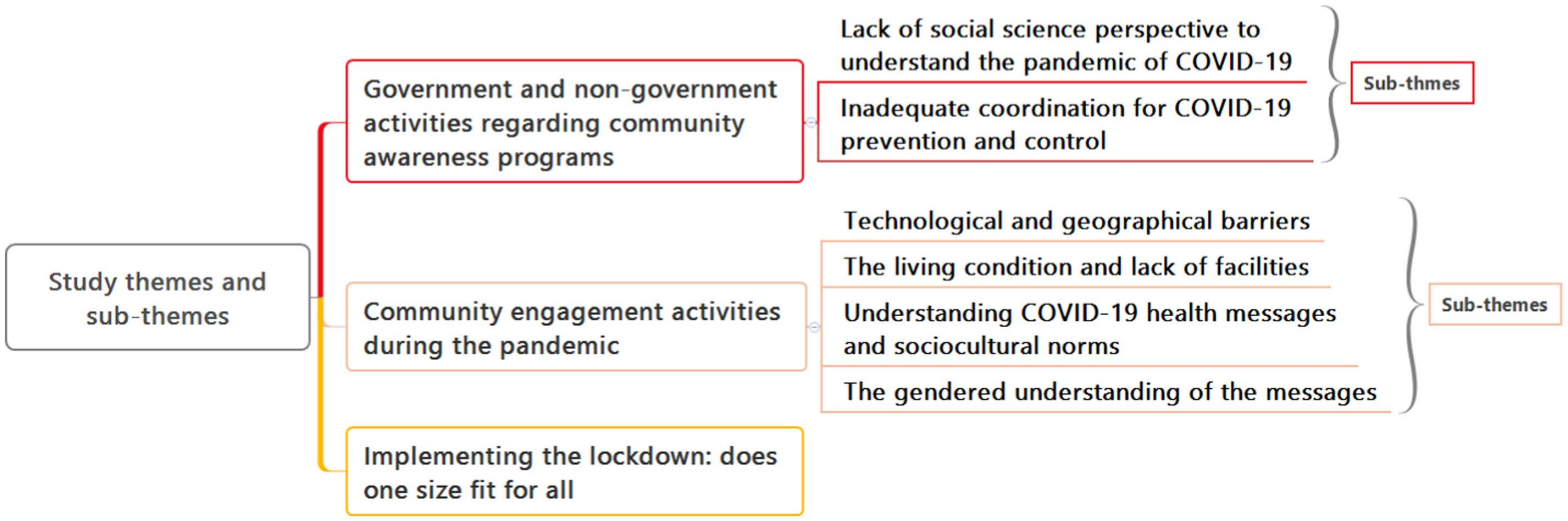
Study themes and sub-themes.

Data analysis followed a rigorous process of thematic analysis (28, 29). Two research assistants transcribed all recorded interviews without participant identification information. The transcripts were then imported into Atlas-ti software version 7.2 for further analysis. SA and MK followed a systematic six-step process, aligned with established guidelines (27). The process commenced with listening to a subsample of transcripts to verify their accuracy and consistency. SA and MK independently coded the data by reading and re-reading the transcripts to ensure the robustness and reliability of the analysis. Subsequently, the two authors engaged in discussions to reach a consensus on common codes and themes. Initially, deductive coding was performed to identify themes based on interview questions. Interviews and group discussions yielded additional themes that were identified inductively. To reach a consensus on the data interpretation, the researchers discussed and compared the similarities and differences of the coded data. Based on their conversations, they compiled a list of shared codes for themes (Figure 2). Most themes from the IDIs, KIIs, and FGDs overlapped across groups and interviews. We have merged themes based on the similarities and differences of the data from the IDIs, KIIs, and FGDs and presented the results in depth.

## Results

### Background information about the study participants

Table 1 presents the socio-demographic information of the participants who participated in the individual in-depth interviews (IDI) and focus group discussions (FGD). The most common occupation among the male participants was day laborer, while most of the female participants were housewives. Some of the male participants had small businesses as their occupation. On average, the participants had three children. Most participants were married, though 12 of the female participants were either divorced or widowed. Most participants had completed education between grades 6 and 10, and 11 participants had completed education beyond grade 11. The average monthly income reported by the respondents was USD 155.

**Table 1:** Social and economic background information of IDI and FGD participants.

Variables	N = 100
Sex	
Male	55
Female	45
The average age of the participants	45
Religion	
Muslim	90
Hindu	10
Years of education	
None	0
1–5	32
6–10	48
11+	20
Marital status	
Married	88
Divorced/widow	12
The average number of children	3
Occupation	
Housewife	35
Small business	30
Day laborer	35
Average income/month (USD)	155

### Theme-1: Government and non-government activities regarding community awareness programs

The first theme presents the community awareness program implemented by the government and non-government organizations. It comprised two sub-themes: (i) Lack of social science perspective to understand the pandemic of COVID-19, and (ii) insufficient coordinated government efforts for COVID-19 prevention and control. The data showed that the government, development partners, and NGOs made tremendous efforts to increase community awareness for the prevention and control of COVID-19. The attempts adopted interpersonal and mass communication approaches to reach communities through social, print, and electronic media. The activities (Table 2) included the distribution of facemasks, the establishment of communal handwashing facilities, and a campaign to maintain respiratory etiquette and social distancing.

**Table 2:** Summary of the “Risk Communication and Community Engagement (RCCE)” messages distributed in the study areas.

Key messages related to RCCE	RCCE activities
Washing hand	With support from development partners, the government’s authorities set up communal hand washing stations in different public places such as the entrance of hospitals, municipality and administrative offices, markets, and bus stations.
Wearing facemask	Messages were communicated through mass media campaigns to wear facemasks to prevent infection. The government and non-government organizations distributed facemasks to the community residents in the study areas.
Maintaining social distancing	Media campaigns and interpersonal communication circulated messages around maintaining social distance and practicing hygiene etiquette.
Lockdown to restrict movement	Mass campaign on ‘stay at home’ and restriction mobility of people by the shutdown of public transportation; also, deployment of law enforcement agencies to ensure restriction of movement during the lockdown.

### Lack of social science perspective to understand the pandemic of COVID-19

All KIIs reported that most measures focused on clinical features of COVID-19: identifying and detecting infectious cases; isolating the cases; calculating the infection rates; establishing hospital facilities to treat infectious patients; and establishing testing facilities. These clinical measures were necessary, but the pandemic was not just a clinical event. It had social dimensions and consequences. The government invested considerable resources to educate people about prevention, but people’s responses to those measures did not meet expectations because of their socio-cultural beliefs about the disease and conditions. In addition, the government formed different technical committees comprised of clinicians and administrative personnel. The participation of sociologists and anthropologists in the committee was not seen as necessary and, hence, the committees were not effective.

### Inadequate coordination for COVID-19 prevention and control

The key informant interviews (KIIs) showed that the implementation of the COVID-19 prevention and control measures in Bangladesh was led by the Ministry of Public Administration despite the Directorate General of Health Services (DGHS) (30) being the responsible authority. The government’s priority area of “risk communication and community engagement (RCCE)” was not adequately addressed due to the absence of a dedicated and functional unit or section to carry out this work. Additionally, the Bureau of Health Education of DGHS—which was primarily responsible for health promotion activities—had limited engagement, as reported by the respondents. The government relied heavily on NGOs for community awareness activities, neglecting its ownership of other critical activities such as testing, treatment, and vaccination. In this regard, one KII said,

*“It would be better to engage or make the Bureau of Health Education a focal for community engagement work, but it did not happen. Still, the Bureau of Health Education is not on board for health education. I think there was a need for a professional approach in the risk communication and community engagement work, but such an approach was absent. Different institutions are working, but those are still scattered and not coordinated.”*

### Theme 2: Community engagement activities during the pandemic

The second theme revealed that mass community awareness activities were conducted but were not successful in engaging communities. The findings showed that community awareness activities were a one-way communication. Information was circulated to the people, but because of several challenges, it was not supplemented with community engagement. One of the challenges was the lack of access by the community to the necessary technology to receive preventive messages disseminated through different media. Geographical barriers, such as remote locations, hindered community engagement efforts. In addition, the population’s living conditions made handwashing and maintaining social distance challenging, restricting people’s ability to adhere to the awareness messages effectively. Furthermore, socio-cultural norms and beliefs influenced the community’s engagement with the prevention activities. This theme covers four sub-themes: technological and geographical barriers, living conditions and lack of facilities, understanding of COVID-19 health messages, socio-cultural norms, and the gendered understanding of the messages.

### Technological and geographical barriers

All the KIIs reported that the awareness activities were primarily conducted in urban areas, which had the highest infection rates. They added that the lack of awareness activities in the rural areas created a belief that the coronavirus did not exist in the villages. A similar opinion was shared by the IDI and FGD participants. They indicated that community awareness activities predominantly focused on urban areas, particularly Dhaka, where urban people could access online information and had greater access to more virtual media. In contrast, rural areas were less familiar with the online awareness activities happening within their localities. The respondents in remote areas in the study districts, such as wetland villages in Kishoreganj, some hilly pockets in Cox’s Bazaar, and riverine villages in Pabna, gained knowledge about awareness campaigns through television and radio. The participants from these rural areas also said they have limited access to social media, and the health workers did not visit their houses to make them aware due to the ongoing movement restrictions.

One KII participant said, *“Our awareness activities at the beginning were in Dhaka and megacities. The infection rate was high in Dhaka, the country’s most populated city. All efforts were made to make aware the city people. It created the misconception that corona does exist in villages. Risk communications and community engagement is not a one-way factor. Communication should be two-way. Like circulating information, the one-way communication was excellent but lacked community engagement.”*

One IDI participant said,

*“Yes, there are many activities in the town. But what about the rural area? Kishoreganj has many villages in the wetlands (haor). These villages are difficult to reach due to their remoteness. I do not think such awareness activities are conducted in the villages.”*

### The living conditions and lack of facilities

All participants shared that they could not practice handwashing, isolation, and social distance as they lived in slums and did not have the required facilities within their homes. They use shared toilets and kitchens with other families. Key informants reported that considering the living and working conditions of the poor people and day laborers, the government and NGOs established communal handwashing stations with soap and water in public places such as bus and train stations, hospitals, and local markets. Awareness activities were conducted by loudspeakers to promote handwashing with soapy water. The IDI and FGD participants reported that the communal handwashing stations were non-functional, and lacked running water or soap. In contrast, the key informants reported that they frequently experienced the stealing of water taps and soap from the public handwashing stations. They also mentioned the budget constraints to keep the communal handwashing stations functional.

### Understanding COVID-19 health messages and socio-cultural norms

The participants in the study had varying beliefs about the effectiveness of facemasks to prevent COVID-19. The IDI and FGD participants knew the importance of wearing facemasks, practicing hand hygiene, and social distancing as key messages to prevent the spread of COVID-19. However, they lacked an understanding of isolation and quarantine measures. The participants appreciated the government’s distribution of free facemasks. However, they only wore them when compelled to do so by law enforcement.

Most participants believed in Allah’s power to create and cure diseases. Thus, if Allah wished them to die from the virus, they could not prevent death by wearing masks and washing their hands. While some believed wearing a facemask could protect them from the virus, others felt masks were only effective if Allah wanted to save them from the disease. Some participants believed the coronavirus spreads through the dust so that a facemask would save them from the virus. In this regard, one respondent said,

*“Allah has ultimate authority over everything, even diseases like COVID-19, and controls life and death. Nothing can save our lives if Allah wants us to suffer from the virus. Whether we wear a mask or not, we will be saved if Allah decides.”*

### The gendered understanding of the messages

The study revealed a gender difference in understanding and practicing the key health messages. Most participants believed that women were less likely to get infected by the coronavirus than men because they stayed home and had more opportunities to clean their hands while performing household chores such as cooking in the kitchen and washing utensils and clothing. Some participants mentioned that women wear hijab, which, in their opinion, was ‘a kind of facemask’ because it covered their face, including their mouth and neck. Because of this view, men did not feel they could infect a woman in the family after returning home from outside. The female study participants said they took no precautionary measures to prevent the virus. This gender dimension further contributed to the weak adherence to “social distancing” in the family—when a husband gets infected, his wife must take care of him, staying closely in the same room. One FGD study respondents said,

*“Women have a low risk of getting infected from coronavirus as they stay home, clean their hands, and frequently work in the kitchen. He added,* “*Women wear hijab, and the hijab is their facemask to protect against coronavirus. Women always clean their hands as they cook and are safer than men.”*

### Theme 3: Implementing the lockdown: does one size fit for all?

All participants in individual and focus group interviews expressed a wish to avoid lockdowns because they would lose income. The study revealed that the IDI and FGD participants strongly opposed the government’s decision to implement lockdowns. Respondents mentioned concerns about their livelihoods and financial stability as their main reasons for disagreeing with lockdowns. The participants believed that prolonged lockdowns would lead to starvation rather than protection from the COVID-19 disease. Participants shared that they faced significant financial difficulties during the lockdown period, which kept them out of work.

Additionally, many respondents reported severe financial hardship and food shortages due to the lockdown. Although the government and NGOs distributed relief, respondents perceived the support (provided in the form of rice, potatoes, pulses, and vegetable oil) as insufficient to meet their daily needs. Consequently, respondents admitted to breaking government regulations, such as movement restrictions, to earn enough money to survive.

One of the IDI respondents said,

*“What will I eat if I stay at home for days? How will my family run without an income? Will the government feed us for months? No. So, I must go out to earn.”*

Another respondent said,

*“I felt like I was in jail during the time of lockdown. I could not work for three months due to the lockdown. It was a horrible situation for my family and me. We suffered from starvation as there was no income.”*

## Discussion

The study’s findings indicate what to do more and how to better promote effective risk communication and community engagement in countries like Bangladesh to prevent and control COVID-19 and other infectious diseases. Community engagement was an essential part of the WHO’s strategy for COVID-19 (7). While this strategy focuses on community empowerment, mobilization, and consideration of local contexts in health care services, the study findings reveal that such focuses needed to be more coherent and better coordinated in Bangladesh. The fragmentation was also apparent in people’s compliance with Bangladesh’s national response plan for COVID-19 and the government’s priority on community participation in healthcare services (13).

The findings critically draw attention to the absence of a public health approach, which had been a central concern in the country’s health systems (31). There is a sharp difference between “health” and “public health.” According to a WHO definition, ‘public health’ is the “art of applying science in the context of politics to reduce inequalities in health while ensuring the best health for the greatest number.” (31) Bangladesh’s National Health Policy 2011 uses the term “public health” to “ensure collective and coordinated efforts of different ministries and divisions and the private sector engaged in public health and medical services.” (32) Krishnan, Kapoor, and Pandav argue that clinicians deal with individuals while public health experts deal with communities, and they emphasize the role of health systems for the prevention and promotion of health (33). Even though COVID-19 was a public health concern, the study found that a public health approach was absent or weak in the COVID-19 awareness activities.

The Ministry of Health and Family Welfare (MoH&FW) is the main agency responsible for delivering health services in Bangladesh. It is responsible for policy planning and implementation, regulating standards, and ensuring people’s health (30). The Ministry also has a community participation approach to health care services in line with the Alma Ata declaration (8). However, a lack of institutional ownership to strengthen community participation and engagement in COVID-19 prevention and control is a concern. The MoH&FW has its community health workforce in a rural setting; however, the whole structure was to deliver health services, not engage the community in the services. This difference between what was done and what should have been done was a severe challenge—a challenge also observed in other studies which argued that community engagement was necessary for better prevention and control of COVID-19 (17).

Bangladesh has made significant economic progress over the years. The World Bank estimated that GDP had increased year-on-year by 1.6% between 1990 and 2019, the last year before the pandemic (34, 35). This economic progress supported the economic transition from a least developed country (LDC) to a Lower-Middle-Income Country (LMIC) (36). Despite the progress, the economic shocks of COVID-19 were severe; many lost their livelihoods, and it became a challenge to implement COVID-19 health guidelines targeting the communities effectively (37). People’s health vulnerability was an issue in the government’s social safety net programs to provide economic support for low-income citizens (38). However, Bangladesh’s National Social Security Strategy had not adequately focused on health emergencies compared with its focus on poverty, climate change, and disaster (39). In the context of a pandemic like COVID-19, the social security strategy must also ensure economic support for the poorer section of people during any community awareness intervention.

Equity and equality are crucial and integral to an effective health system response to a pandemic. Bangladesh’s national health policy explicitly focuses on health equity and equality (32). Different operational plans and strategies of Bangladesh’s health services authority emphasize the needs of poor, vulnerable, and marginalized groups of people in health services delivery (40). However, our results showed that the COVID-19 awareness activities were biased toward urban areas while the hard-to-reach areas were left out. Women and people in hard-to-reach areas had less access and greater difficulty accessing health services and this disparity intensified during the pandemic (41). This empirical outcome stands in sharp contrasts to the MoH&FW’s health policy and the gender equity strategy, which aim to ensure health equity and gender-responsive health services delivery in the country (42). We would argue that community awareness interventions on health matters should address this (in)equity and (in)equality in reaching the people. This is particularly important when one’s vulnerability in a pandemic like COVID-19 goes beyond biological issues and involves socioeconomic issues as well. Any public health intervention, including the Social and Behavioral Change Communication (SBCC) campaign, should consider this broader aspect for its maximum reach and coverage.

Our findings, importantly, draw attention to the notion of “info-demic” as the people in different study areas understood COVID-19 health messages differently (43). WHO used the term “info-demic” to describe the situation of misinformation during COVID-19, which led to confusion, panic attacks, and anxiety among the people (44). Such misinformation is uncontrolled and a barrier to implementing policies and guidelines during a pandemic (43). This info-demic is rooted in people’s socio-cultural beliefs. The study findings revealed that the people received and interpreted the COVID-19-related information in various ways. It often involved people’s cultural beliefs, such as connecting them to their religion (Islam), particularly the “power of Allah.”

The paper provides lessons for future community awareness design issues in a pandemic like COVID-19 in a country like Bangladesh. The lessons include an- equal emphasis on disseminating information and ensuring a common understanding of the information; capitalizing on community strength; utilizing various community-based platforms for health education; increasing focus on the hard-to-reach areas; strengthening and continuing collective and collaborative action; considering socioeconomic situations of the target vulnerable populations; and integrating cultural perspectives into community awareness intervention.

## Limitation

The study had three notable limitations. Firstly, the study was conducted in specific areas of Bangladesh, and the findings may not be generalizable to other regions. Secondly, the study did not assess the *effectiveness* of community awareness interventions in preventing or controlling COVID-19. Finally, the study did not explore the impact of the pandemic on other health issues and the healthcare system in Bangladesh. Despite these limitations, the study provides valuable insights into community engagement and risk communication strategies in the context of a pandemic like COVID-19 in Bangladesh.

## Conclusion

The discussion critically analyses the study’s findings on community engagement and public health approach in COVID-19 prevention and control in Bangladesh. It highlights the fragmentation in the community empowerment and mobilization strategy and the absence of a public health approach in COVID-19 awareness activities. The discussion also emphasizes the need for institutional commitment to strengthen community participation and engagement in COVID-19 prevention and control and the role of socioeconomic factors, equity, and equality in the health system response.

The discussion further underscores the importance of considering socio-cultural beliefs and integrating cultural perspectives in community awareness interventions to address the “info-demic” in a pandemic like COVID-19. It is crucial to consider different needs, contexts, and priorities when developing public health interventions and policies related to pandemic control. A one-size-fits-all approach may not effectively address various communities’ diverse challenges and experiences. The discussion concludes by providing future design issues for community awareness in a pandemic, including a common understanding of information, capitalizing on community strength, utilizing community-based platforms for health education, focusing on hard-to-reach areas, continuing collective and collaborative action, and integrating cultural perspectives into community awareness interventions.

The paper also calls for more research to be done from a public health perspective, to learn more about how the government of Bangladesh’s institutions can work together better to make its community participation approach to health service delivery effective. It is important for a community-based health system to be resilient to prevent future pandemics and communicable diseases.

## Data availability statement

The original contributions presented in the study are included in the article/supplementary material, further inquiries can be directed to the corresponding author.

## Ethics statement

The Ethical Review Committee (ERC) of the International Centre for Diarrhoeal Disease Research, Bangladesh (ICDDR,B) reviewed and approved the study proposal (PR-21135). The study adhered to the ethical guidelines of the authors’ institution for research. Participants provided informed written consent following the regulations of the ERC. The participants agreed to publish data from the interviews in a non-identifiable form.

## Author contributions

MK: Conceptualization, Data curation, Formal analysis, Funding acquisition, Investigation, Methodology, Project administration, Resources, Supervision, Writing – original draft, Writing – review & editing. AR: Conceptualization, Data curation, Formal analysis, Methodology, Project administration, Writing – original draft, Writing – review & editing. DR: Project administration, Writing – review & editing. SA: Funding acquisition, Supervision, Writing – original draft, Writing – review & editing.
